# Encapsulated Papillary Carcinoma of the Male Breast: A Case Report and Literature Review

**DOI:** 10.7759/cureus.93922

**Published:** 2025-10-06

**Authors:** Kristina Anichkina, Alina Pasternak, Gurami Kvetenadze, Evgenii Shivilov, Tamara Pavlova

**Affiliations:** 1 Oncology, Loginov Moscow Clinical Scientific Center, Moscow, RUS; 2 Oncology, Federal State Budgetary Educational Institution of Higher Education, “Russian University of Medicine” of the Ministry of Health of the Russian Federation, Moscow, RUS; 3 Oncology, Central Research Institute of Radiation Diagnostics, Moscow, RUS

**Keywords:** diagnosis of papillary carcinoma of the male breast, encapsulated papillary carcinoma of the male breast, incidence of papillary carcinoma in men, intracystic papillary carcinoma of the male breast, male breast cancer, surgery of papillary carcinoma

## Abstract

This case report aims to demonstrate the challenges associated with the diagnosis and treatment of papillary carcinomas of the male breast. Based on a comprehensive screening, which included a pathoanatomic examination of the surgical material after a simple Pirogov mastectomy (a surgical procedure where all or part of a breast and axillary lymph nodes are removed), the diagnosis was confirmed as encapsulated papillary cancer of the left breast. In the immunohistochemical study, the estrogen receptor was 100%, the progesterone receptor was 90%, and Ki-67 was 60%. Therefore, the patient was prescribed adjuvant hormone therapy with tamoxifen. There are no specific diagnostic clinical, imaging, or core needle biopsy features of encapsulated papillary carcinoma of the male breast. This often leads to more radical surgery for this low-grade neoplasm. Before choosing the extent of surgery, it is necessary to consider the primary size of the tumor along with an intraoperative assessment of the resection margins.

## Introduction

Male breast cancer is an uncommon malignancy, and papillary carcinoma represents a particularly rare histological subtype, often posing significant diagnostic and therapeutic challenges. We present the case of a 66-year-old male who self-detected a palpable mass in his left breast. Clinical examination, mammography, and ultrasound revealed a large, heterogeneous encapsulated lesion suspicious for malignancy. Core needle biopsy suggested non-invasive papillary carcinoma with strong hormone receptor expression. The patient underwent a simple Pirogov mastectomy, and histopathological analysis confirmed encapsulated papillary carcinoma without evidence of invasion or nodal involvement. Immunohistochemistry demonstrated estrogen receptor positivity (100%), progesterone receptor positivity (90%), and a proliferative index (Ki-67) of 60%. Adjuvant endocrine therapy with tamoxifen was initiated.

This case highlights the absence of distinctive clinical, radiological, or biopsy features of encapsulated papillary carcinoma in men, which frequently results in radical surgery despite the tumor’s low-grade nature and favorable prognosis. Current evidence suggests that organ-preserving procedures may offer equivalent oncological safety, provided tumor size and resection margins are carefully assessed intraoperatively. A review of the literature confirms that male papillary carcinoma is typically hormone receptor-positive, rarely metastasizes, and carries an excellent long-term survival rate. Recognition of its clinicopathologic profile is essential to avoid overtreatment and optimize individualized management strategies in this rare entity. This case report aims to demonstrate the difficulties of the diagnosis and specific treatment of male papillary carcinoma using a clinical example.

## Case presentation

**Figure 1:**
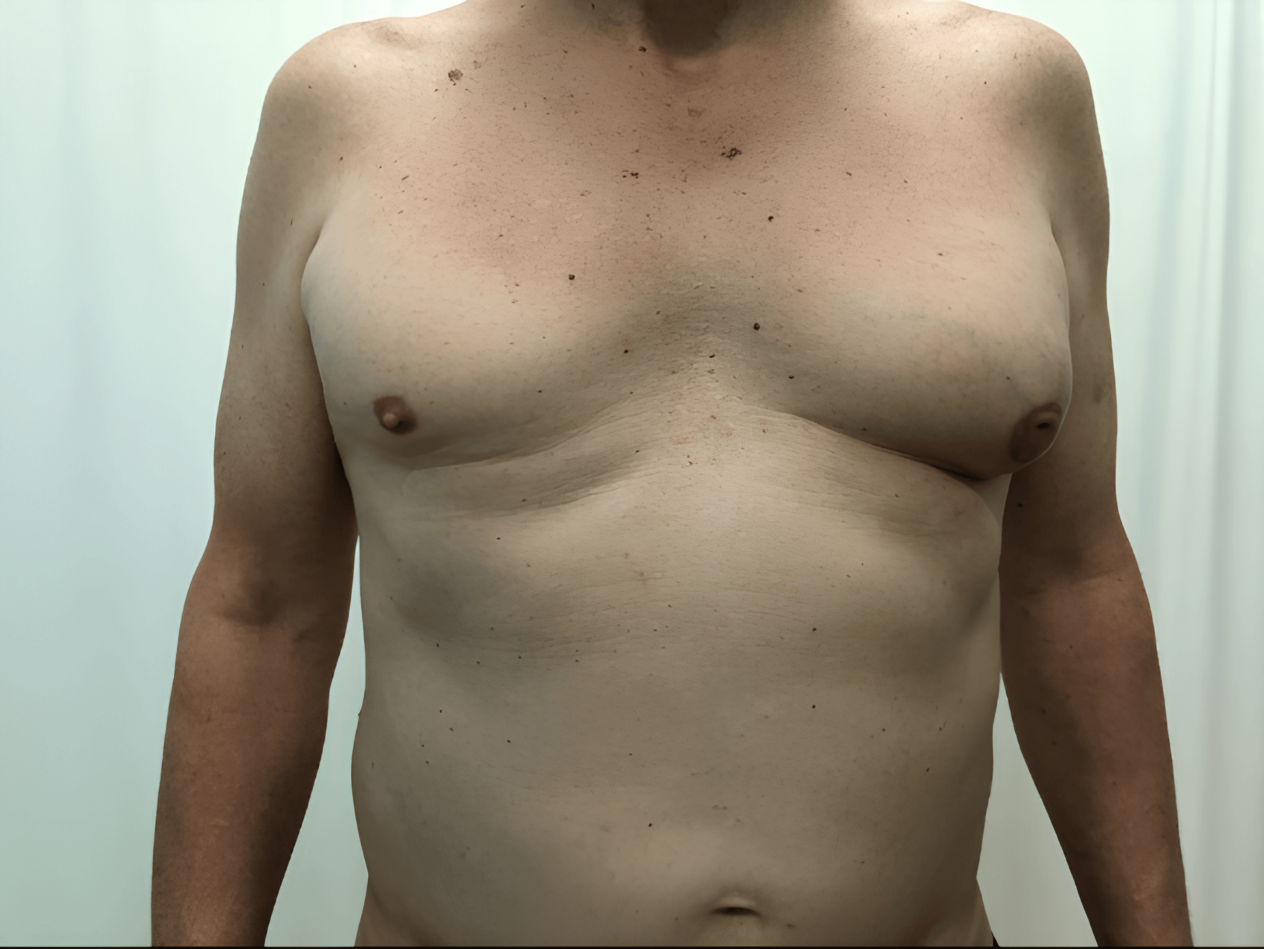
Appearance of patient S. Asymmetry of the mammary glands (S > D) and changes in the left nipple-areolar complex are noted.

Patient S, 66 years old, independently discovered a mass in his left breast in 2021, after which he went to a specialized institution, where he underwent a full diagnostic complex, including clinical examination, X-ray mammography, breast ultrasound, and trephine biopsy. On physical examination, the mammary glands were asymmetrical; the right one was featureless, and the left one was significantly enlarged in the central part due to a mass. Palpation of the left breast revealed a nodular mass of dense consistency, with clear contours, mobile relative to the chest, and measuring 5 × 5 cm in size. The nipple was tightened and covered with a serous crust. Regional lymph nodes were not enlarged (Figure 1).

**Figure 2:**
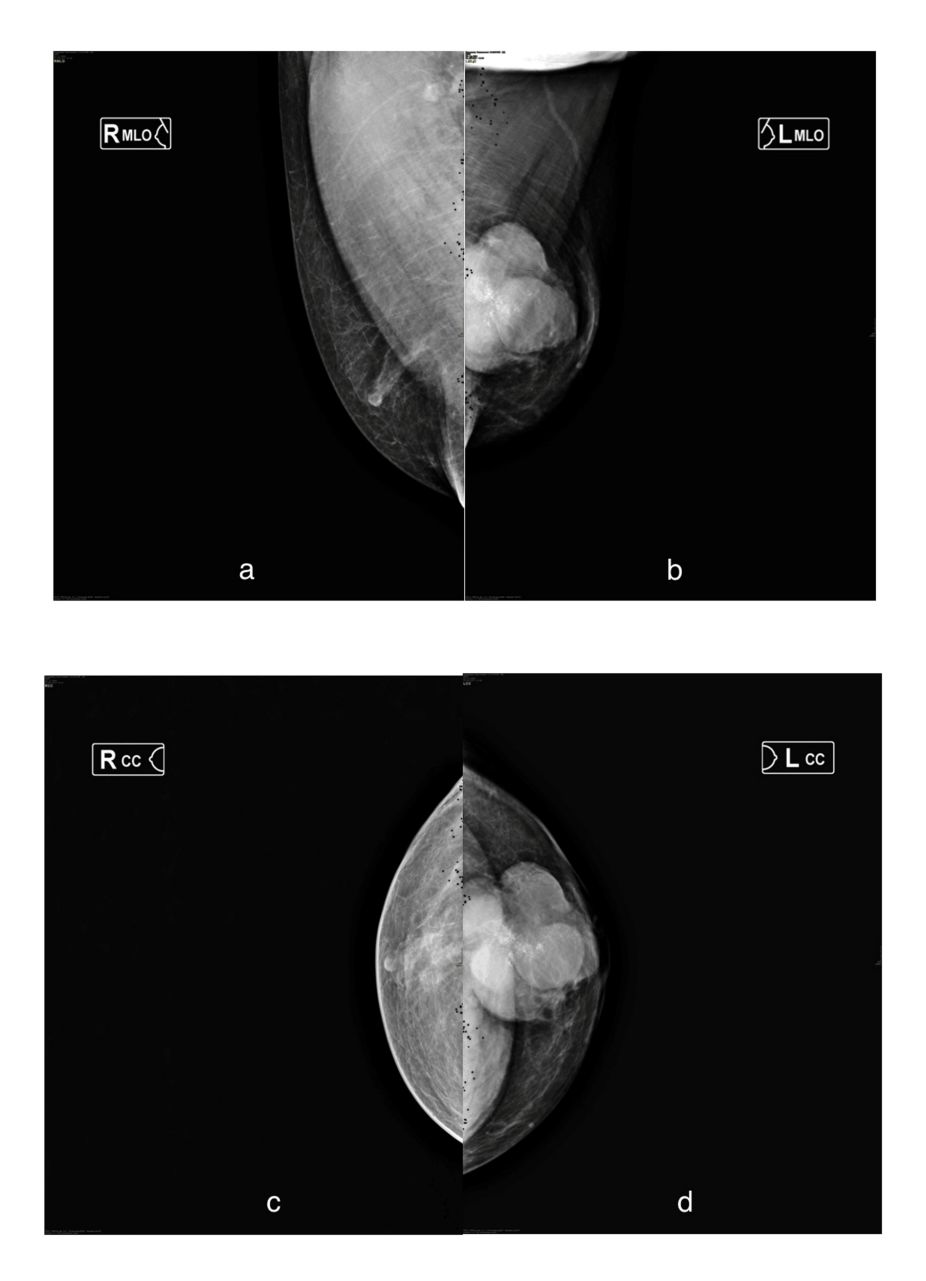
Review mammograms of patient S performed in two standard projections. (a, c) The right mammary gland in the mediolateral and craniocaudal projections. (b, d) The left mammary gland in the mediolateral and craniocaudal projections; in the central part of the left mammary gland, a multinodular mass of increased intensity with a lumpy contour of heterogeneous structure, with inclusion of multiple pleomorphic calcinates up to 2.5 mm in size, is visualized.

According to the results of review X-ray mammography performed in two standard projections in the left breast, a multinodular mass of increased intensity with a lumpy contour of heterogeneous structure, with inclusion of multiple pleomorphic calcinates, occupying one third of the gland, measuring 66 × 68 × 61 mm, was centrally visualized. The detected changes were determined to be suspicious for breast cancer (BIRADS category 4c) (Figure 2).

**Figure 3:**
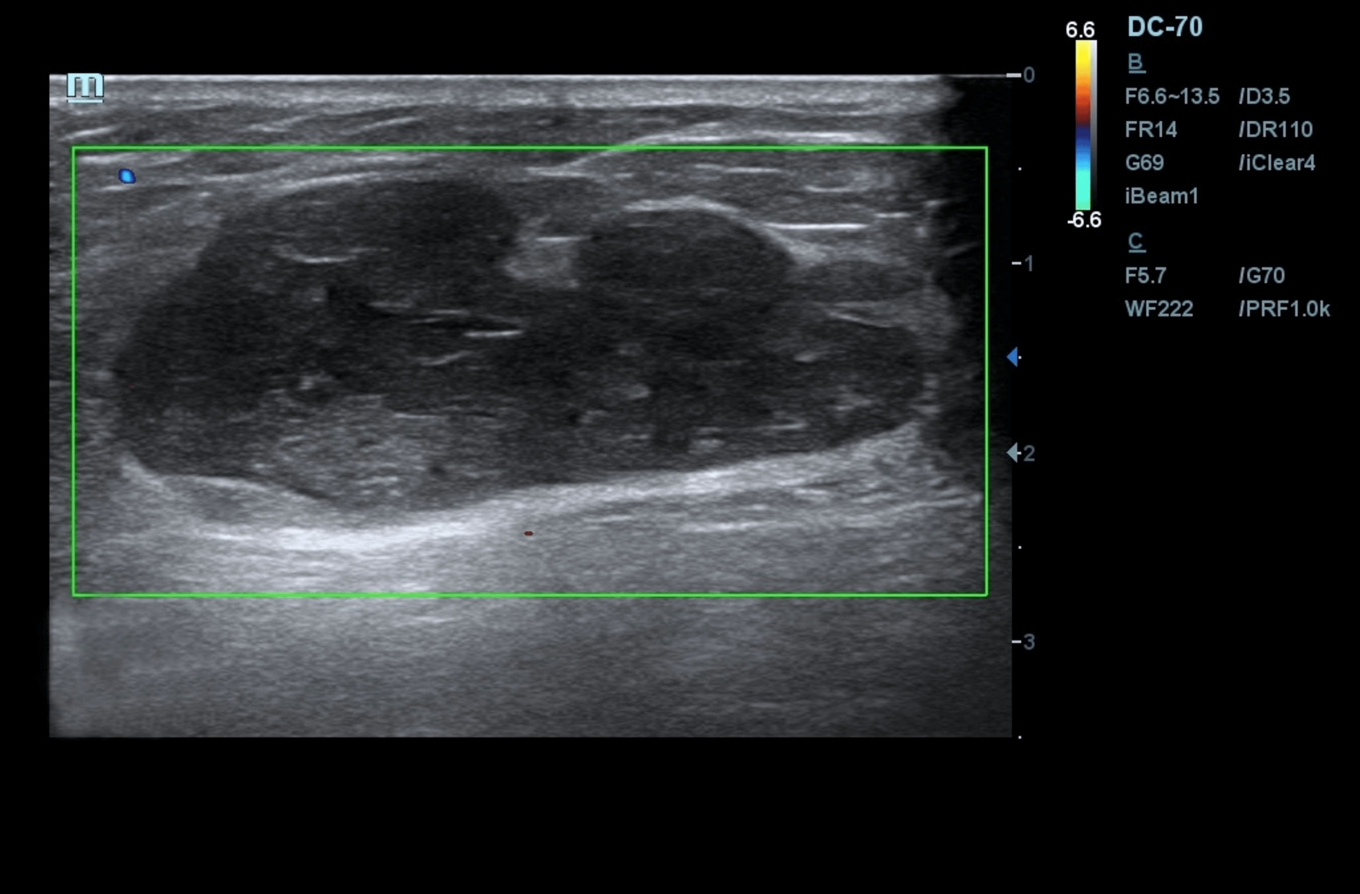
Sonogram of patient S in standard B-mode. Performed in two standard projections: in the central part of the left breast, an encapsulated volumetric nodular neoplasm of irregular oval shape of heterogeneous echostructure with anechogenic and hyperechogenic inclusions of BIRADS 4c category can be visualized.

During ultrasound examination of mammary glands and regional lymphatic outflow zones on an expert-class device, equipped with a linear scanning transducer (7-15 mHz) in the standard B-mode, an encapsulated volumetric nodular mass of irregular oval shape of heterogeneous echostructure with anechogenic inclusions and single loci of perinodular blood flow (BIRADS 4c) was centrally visualized on the left side, exceeding the transducer aperture and intact in relation to the skin and the edge of the pectoralis major muscle. No changes in regional lymph nodes were detected (Figure 3).

**Figure 4:**
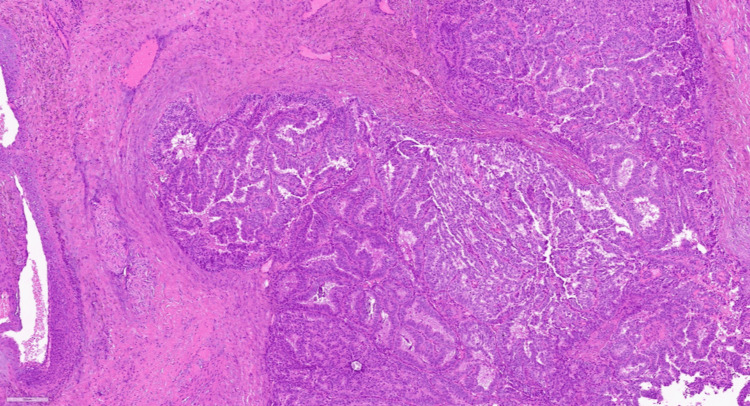
Histologic picture of the breast biopsy. Fragments of fibrous tissue with lymphocytic-histiocytic infiltration with the presence of hemosiderophages. Outside the stroma, multiple papillary structures represented by fibrovascular rods covered with epithelial cells with eosinophilic cytoplasm with hyperchromic and moderately polymorphic nuclei, with signs of proliferation and formation of solid, cribrotic structures with the presence of microcalcinates.

**Figure 5:**
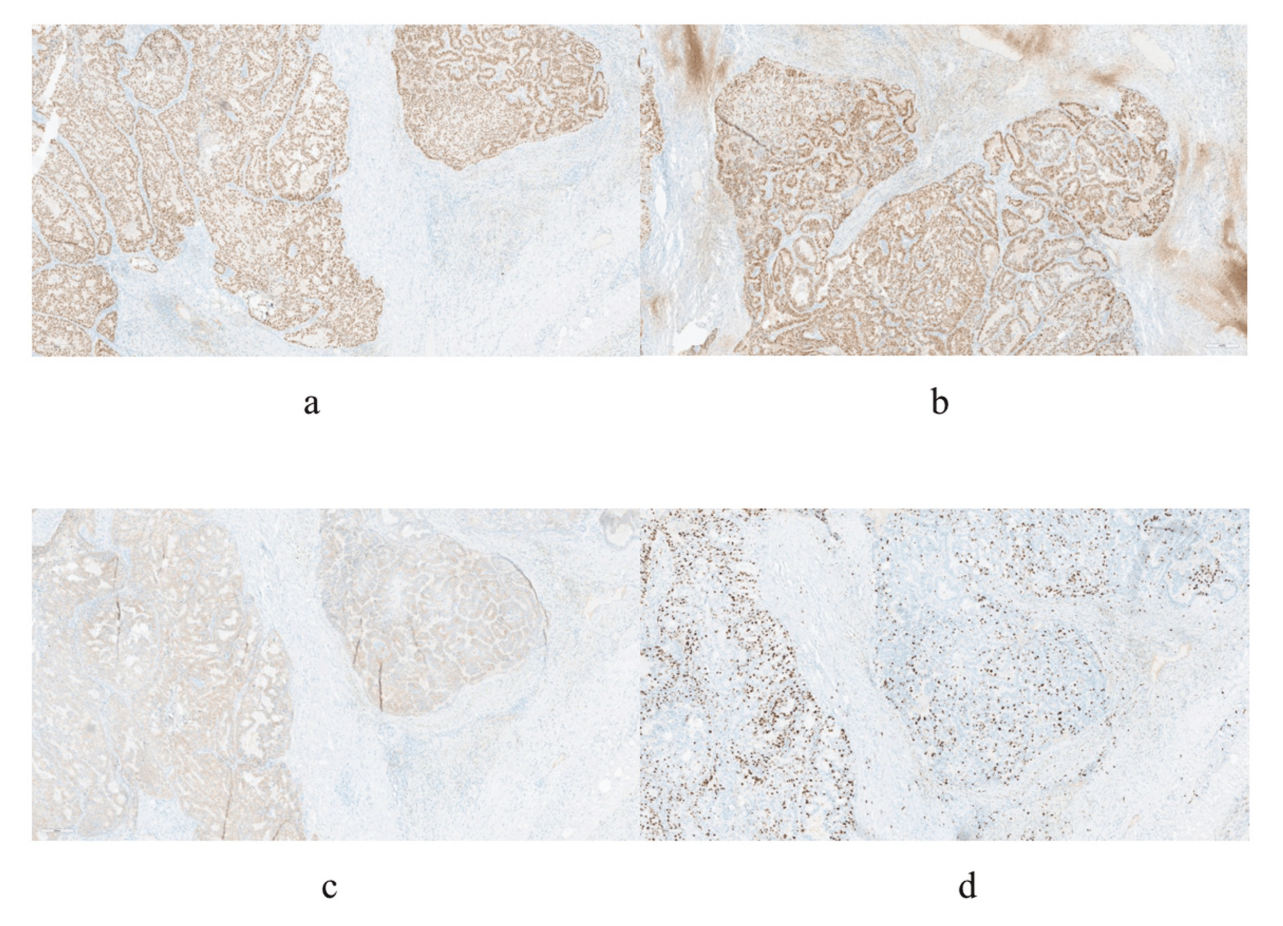
Immunohistochemical picture of the tumor. (a) Estrogen receptors (positive expression). (b) Progesterone receptors (positive expression). (c, d) Ki-67 expression in 60% of tumor cell nuclei, magnification ×100.

After a baseline breast radiological examination, the patient underwent a core needle biopsy of a left breast mass under ultrasound navigation, the results of which verified non-invasive papillary carcinoma, with positive expression of estrogen and progesterone and absence of a layer of myoepithelial cells around the tumor structures (Figures 4, 5).

**Figure 6:**
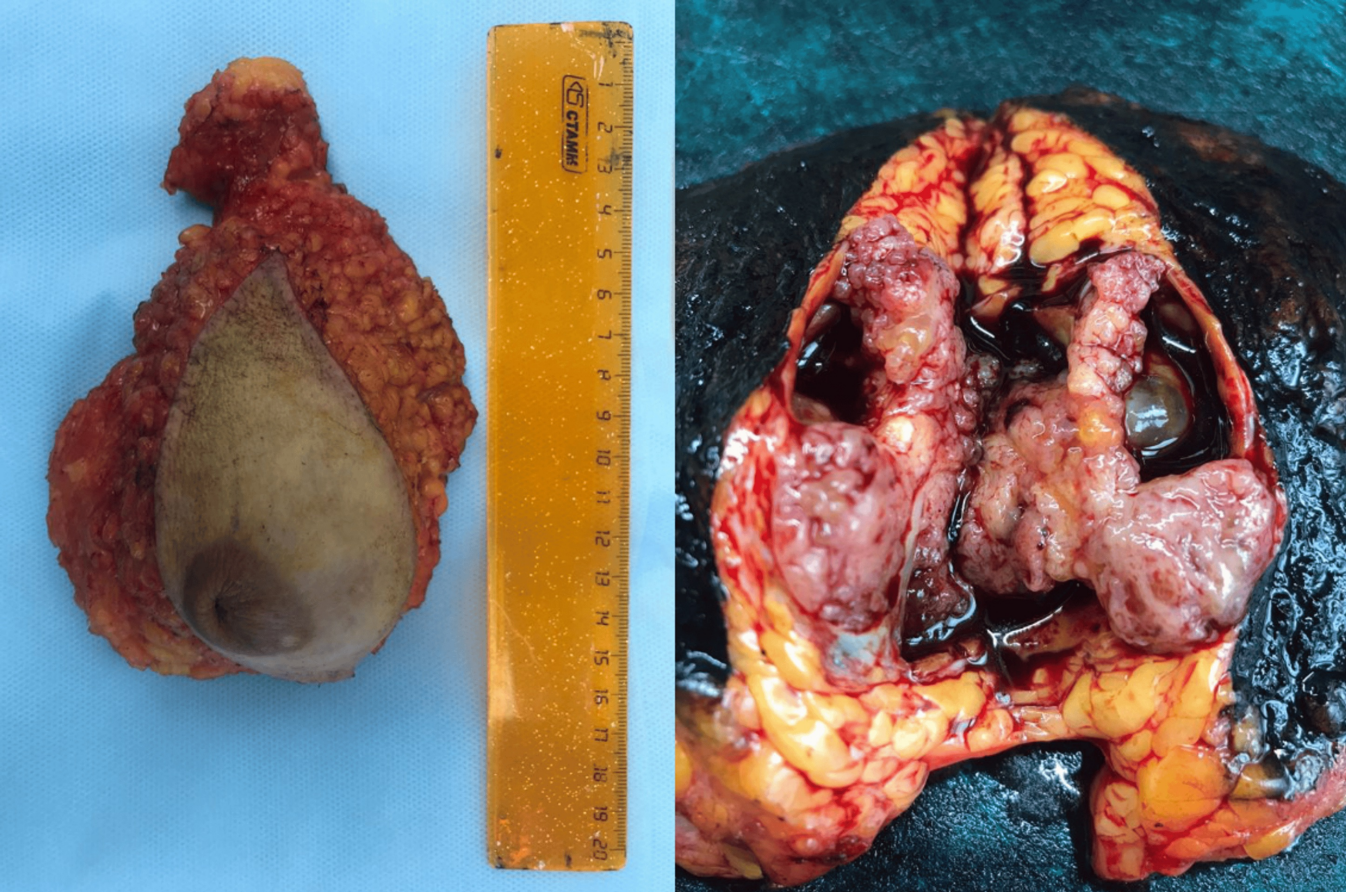
Pathological examination of the postoperative material.

After a comprehensive examination, no data on the prevalence of the pathologic process were obtained. According to the decision of the oncologic consilium at the first stage of treatment, the patient underwent a simple mastectomy according to Pirogov on the left side. On the pathologoanatomical examination of the operative material, the sample size was 15 × 13 × 4 cm, and the tumor size was 5 × 4 × 4 cm. No reliable signs of invasive growth were found. There was also no tumor growth along the resection margin (Figure 6). The diagnosis was intraductal encapsulated papillary cancer of the left breast. On immunohistochemical examination, estrogen receptor was 100%, progesterone receptor was 90%, and Ki-67 was 60%. The patient was prescribed adjuvant hormonal therapy with tamoxifen (Figure 6).

## Discussion

Papillary neoplasms are a heterogeneous group of breast diseases that include intraductal papilloma, papilloma with atypical ductal hyperplasia, ductal carcinoma in situ, and intracystic and intraductal papillary carcinoma [1]. Encapsulated papillary carcinoma is morphologically similar to intraductal papillary carcinoma, except that myoepithelial cells are absent from the surrounding thick fibrous capsule [2]. The demonstrated clinical example shows the absence of a specific clinical and instrumental picture of papillary carcinoma. In the following comparative analysis of data from international publications, all operations aimed at complete excision of the surrounding tissues (Table 1). However, it is possible to perform not only radical mastectomy but also organ-preserving surgery, as the prognosis of this tumor is a 92.0% recurrence-free survival rate after 10 years in case of local recurrence and distant metastasis [3]. Regional lymph nodes are rarely affected [4,5]. Wang et al. (2016) reported that the incidence of regional metastases was 2.7% [6]. Therefore, the need for axillary lymphodissection and sentinel lymph node biopsy remains an open question. In studies conducted by Tang (et al.) and Jackson (et al.) reported an excellent prognosis confirmed by a significant recurrence-free survival in patients with encapsulated papillary carcinoma without invasion at 95.8% and 96.7%, respectively [7,8]. According to international publications, there were no signs of their involvement in the pathologic process when the sentinel lymph nodes were biopsied [5,9-11]. In eight scientific papers, patients were offered an anti-estrogen drug (tamoxifen), taking into account the positive status of hormone receptors [2,3,12-17]. The clinical case reported in this article also used tamoxifen as endocrine therapy. It is believed that postoperative adjuvant therapy for breast cancer in men should be consistent with the treatment of this disease in women [18]. In addition to endocrinotherapy, radiation and chemotherapy may be used in postoperative treatment for advanced tumor processes [2,13,14,19]. Zhang et al. in 2018 described the prognosis of encapsulated papillary tumor, indicating that the recurrence-free survival rates were 99.1%, 97.2%, and 92.0% after 2, 5, and 10 years, and the overall survival rates were 98.9%, 92.2%, and 85.6%. Only three patients were found to have recurrences due to large tumor size and failure to perform intraoperative exploration of the resection margins [20]. Encapsulated intracystic tumor is considered a slow-growing form of invasive carcinoma with a favorable prognosis (10-year recurrence-free survival rate was 92.0%) [20]. These tumors have a low incidence of regional recurrence and distant metastases (average of 6.1%) [4,21,22].

**Table 1:** A comparative analysis of international publications on detected papillary carcinomas in men. PC = papillary carcinoma; EPC = encapsulated papillary carcinoma; MRM = modified radical mastectomy; RM = radical mastectomy; SLNB = sentinel lymph node biopsy; RT = radiotherapy; HT = hormone therapy

Authors	Number of patients	Age	Visualization ultrasound/mammography	Results of preoperative imaging	Preoperative biopsy results	Type of surgery	Axillary area	Adjuvant therapy
Al Salloom, 2015 [15]	1	53	No/Yes	No information	EPC	MRM	Axillary dissection	No information
Banys-Paluchowski et al., 2016 [13]	1	62	Yes/No	Large irregular structure	EPC	RM	Axillary dissection	HT, RT
Hu et al., 2016 [17]	1	59	Yes/Yes	Complex cystic mass with a solid component	PC	RM	SLNB	HT
Agrawal et al., 2017 [23]	1	52	Yes/No	A faintly delineated homogeneous mass	No information	RM	No information	No information
Kumar et al., 2017 [24]	1	53	No information	No information	Infiltrating ductal carcinoma	MRM	Axillary dissection	No
Kinoshita et al., 2018 [2]	1	64	Yes/No	Solid cystic mass	Variegated cells with an enlarged oval node	RM	SLNB	HT
Yilmaz et al., 2018 [22]	1	63	Yes/Yes	Solid cystic mass	EPC	RM	SLNB	No information
Stolnicu et al., 2018 [25]	1	75	No information	No information	Papillary cells with minimal atypia	RM	SLNB	No information
Mok et al., 2018 [5]	1	89	Yes/Yes	Solid cystic mass	No information	RM	SLNB	No
Akin et al., 2019 [26]	1	72	Yes/Yes	Complex cystic mass with a solid component	EPC	RM	No	No
Esposito et al., 2019 [27]	1	56	Yes/Yes	Cystic mass	EPC associated with multiple foci of ductal carcinoma in situ	RM	SLNB	HT
Singh et al., 2020 [14]	1	40	Yes/No	Cystic lesion with papillomatous formation	EPC	RM	SLNB	HT
Hassan et al., 2020 [28]	3	Average of 61 years	Yes/Yes	1: complex cystic mass with a solid component. 2: distinct hypoechogenic area. 3: well-defined complex lesion	Two patients: EPC. One patient: no information	Three patients: RM	Two patients: SLNB. One patient: no	No information
Luo et al., 2020 [16]	3	Average of 71 years	Yes/Yes	1: complex cystic mass with a solid component. 2: distinct hypoechogenic area. 3: well-defined complex lesion	Two patients: EPC. One patient: no information	Three patients: RM	Two patients: SLNB. One patient: no	Two patients: HT. One patient: No
Avau et al., 2021 [3]	1	46	Yes/Yes	On the right, an irregular, lobulated cyst; on the left, a complex cyst	Invasive ductal carcinoma on the right and two encapsulated papillary carcinomas associated with foci of ductal carcinoma in situ on the left	Bilateral RM	Bilateral SLNB	HT, chemotherapy, axillary RT
Huang et al., 2022 [19]	1	63	No information	No information	Invasive carcinoma	MRM	Axillary dissection	Chemotherapy
Li et al., 2022 [29]	1	62	Yes/Yes	Complex mass	Fat necrosis, PC	RM	SLNB	No
Johnson et al., 2015 [30]	1	70	Yes/Yes	Complex cystic mass with a solid component	PC	RM	SLNB	No information
Present case	1	66	Yes/Yes	Heterogeneous mass with anechogenic inclusion and solid component	PC	RM	No	Hy

## Conclusions

There are no specific clinical and imaging characteristics of encapsulated papillary carcinoma in men. The primary tumor size and the presence of intraoperative evaluation of the resection margins should be considered when selecting the extent of surgical intervention. Organ-preserving operations have the same oncologic safety as radical mastectomies. Papillary carcinoma in men has a favorable prognosis (rarely metastasizes and recurs).
